# Real-time data visualization of welding robot data and preparation for future of digital twin system

**DOI:** 10.1038/s41598-024-59146-9

**Published:** 2024-05-03

**Authors:** Péter Magyar, János Hegedűs-Kuti, József Szőlősi, Gábor Farkas

**Affiliations:** 1 Doctoral School of Informatics, Eötvös Lóránd University, Budapest, 1117 Hungary; 2 Savaria Institute of Technology, Eötvös Lóránd University, Szombathely, 9700 Hungary; 3 Department of Computer Algebra, Eötvös Lóránd University, Budapest, 1117 Hungary

**Keywords:** Mechanical engineering, Computer science

## Abstract

The application of industrial technologies is undergoing significant changes. Finding the level at which to use efficient cyberphysical systems is perhaps one of the most important technical preparatory tasks in implementing digital manufacturing. Welding technology systems are investigated, and a framework for capturing the data sets required for data-driven manufacturing is developed. To make full autonomy in a manufacturing environment meaningful, formerly isolated groups of equipment need to be organized into a production information system. In our research, a test system is created that can implement a digital virtual interface and achieve new levels of efficiency with a future digital twin system. In the discourse of the study, the technological parameters of welding test pieces were investigated, namely the available measurement data sets of current, and voltage data. In the summary section, most of the tasks and research directions are presented, which can be envisaged as a continuation of the present study. Our study will be followed by further research, already testing a complete digital twin system, thus reaching another milestone on the way to autonomous manufacturing.

## Introduction

**Figure 1:**
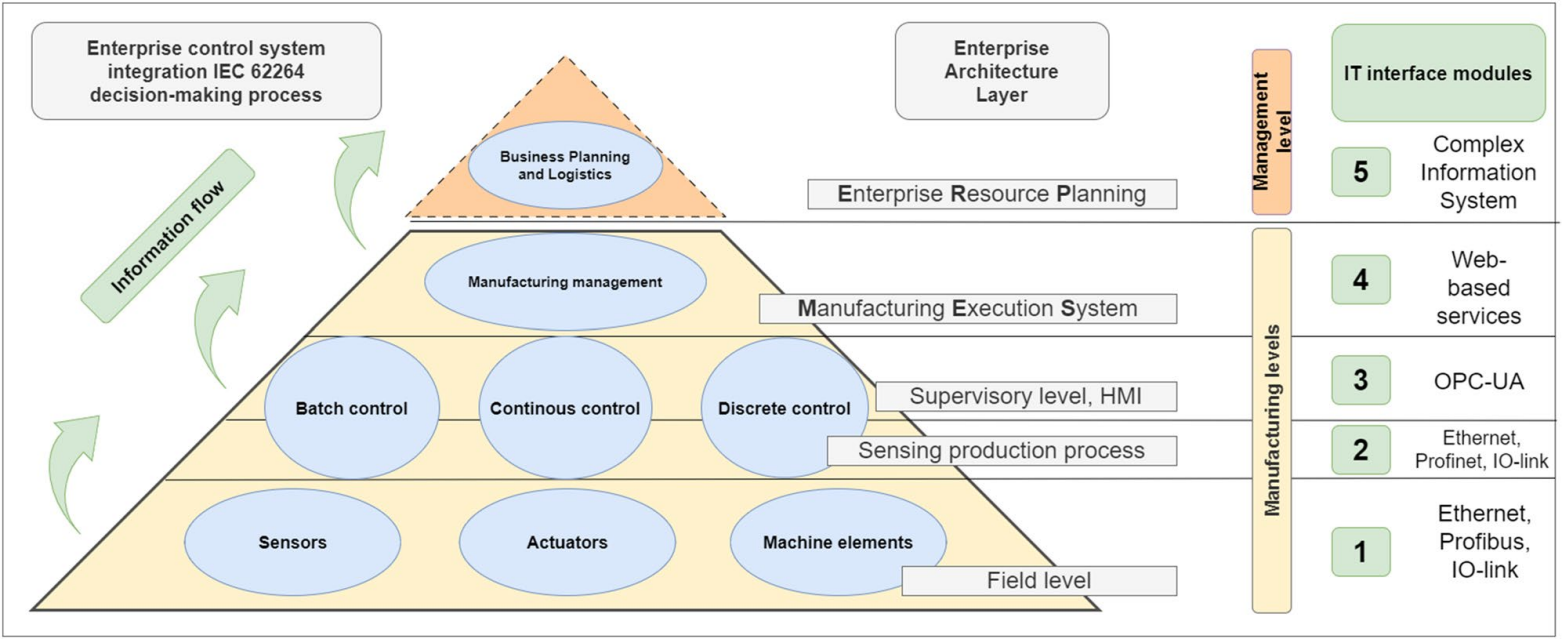
Pyramid hierarchy of a manufacturing system according to IEC 62,264 system description.

The manufacturing needs of the different manufacturing systems are very diverse. The economically remarkable results that have been continuously achieved since the second half of the last century with the development of cyber-physical systems require an advanced IT (Information Technology) subsystem and toolbox^1^. It could be said that the development and support of computer technology^2^ has led to unprecedented production efficiencies, the results of which were published in 1985 in several studies. This was the early CIM (Computer Integrated Manufacturing) system. It was shown that without integrating computer science tools into engineering subsystems, such efficiencies could not be achieved in isolated groups of machines. With the extraordinary advances in computer science, it is possible to build a capable subsystem to support each engineering subsystem^3–5^. Data-driven manufacturing is created according to the levels of the automation pyramid based on the IEC 62264–1:2013 specification^6–8^. The relevant levels of the structure are shown in the Fig. 1 below. The field devices at the bottom of the pyramid provide information that can trigger a decision-making mechanism at the top of the pyramid. The system is based on ERP (Enterprise Resource Planning)—MES (Manufacturing Execution System) communication, i.e. the individual components of the complex software ensemble effectively support the business modules.

**Figure 2:**
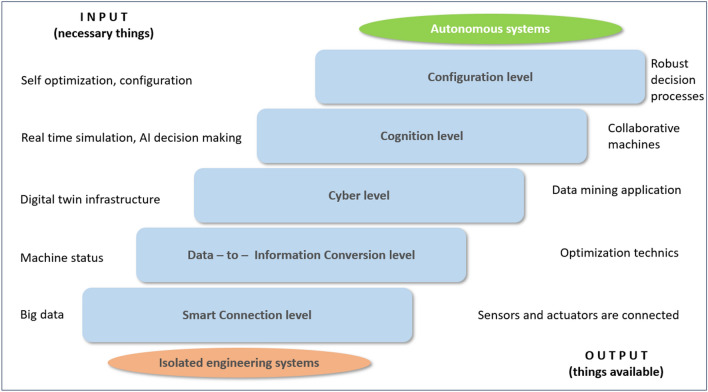
The path to autonomy according to 5C architectural models^12^.

It has been focused on the production system, so the area of the ERP, i.e. the top of the pyramid, is marked with a dashed line in our diagram. Meanwhile, the associated interfaces of the industrial IoT (Internet of Things) help to manage IT and engineering applications as a single system^9^. Gathering data and with it, obtaining relevant data from a smaller set is key to making the right decisions. But to create a fully autonomous system, it is necessary to think in terms of an evolutionary path. The following Fig. 2 shows the individual process steps^10,11^.

As shown in the figure, the whole horizon of complex system design can be seen, i.e. moving from the use of isolated groups of machines towards a complex system capable of autonomous decision-making. At the first stage, it is already necessary to start data collection, the milestone output of which is the existence of the associated data-providing field tools. At the subsequent stages, the technological level is higher, which may already cause problems in the industrial sectors as a whole, since the speed of system implementation varies across the entire technological vertical. A major challenge for the future is to assess and operate these entities at a common autonomous level of development^13,14^.

### IT components of production systems

Achieving autonomy requires the simultaneous development of several components. If welding manufacturing is considered more strictly among the manufacturing technology systems, these components could be a document management module designed to manage complex information system standards and directives. There are two ways to approach the operation of this module from the IT side. There are those documents that come from external providers, for which a web-based solution, such as a service-oriented architecture-based web application, may be appropriate. At the same time, the production process generates a sufficient number of documents to guarantee the quality of the production. These should be managed through a database management system. However, in all cases, the right data science tool will help to visualise the data generated at the bottom of the production system to support the production mechanism at the top of the system. The production system is supported by various neural networks that predict, for example, the correct choice of technical parameters. In the same way, it would make sense to use algorithms to control the production system in the knowledge of the different production events, so that the yield shows the highest efficiency.

The best way to do this is to think about a digital twin application in the future. The wording of a digital twin can often be confusing in terms of the information available. Many people know it mostly as a simulation tool, but this is a flawed approach because simulation as geometric feedback has been used in CNC (Computer Numerical Control)-controlled systems since the mid-1980s. At the next conceptual level, the digital twin is identified as a digital copy of the real physical system, which virtually follows the behaviour of the physical system. However, in our view, the digital twin is a complex system based on optimisation, where it is true that it displays data characteristic of the production in the space virtually followed by the physical system, algorithms process the data in the background and then feed back the results of the processing to the physical system, i.e. its technological parameters^15–17^.

Creating such a system is a very complex task, it must be said that the system design must first be approached at the application development level. Then, by running the applications in a simple framework, the whole structure can be continuously improved. Our study is an attempt to develop a digital interface and to show the relevant data. Moreover, we outline a plan for how the research can be developed further.

## Methodology

The framework, which uses a digital twin to present the current welding robot movement and record the motion and welding parameters—for parameter estimation, real-time system monitoring, seam sequence optimization will be fine-tuned^18–22^. The ideal weld parameters will also be supported by mathematical modelling^23,24^. The framework will become complete when the optimal values are proposed, and autonomous when adapted. In addition to the real welding of the parts, the virtual robot is working in parallel, which is monitored on the connected computer. The extended framework now includes a comparison with the recommended values and recommendations, which will be detailed in a future paper. The creation of the digital twin starts by detecting the connectivity options and activating them. The connection consists of two parts, firstly the control of the robot and secondly the connection of the power supply of the welder to the test system.

### Connecting the robot controller and the power source in the manufacturing system

**Figure 3:**
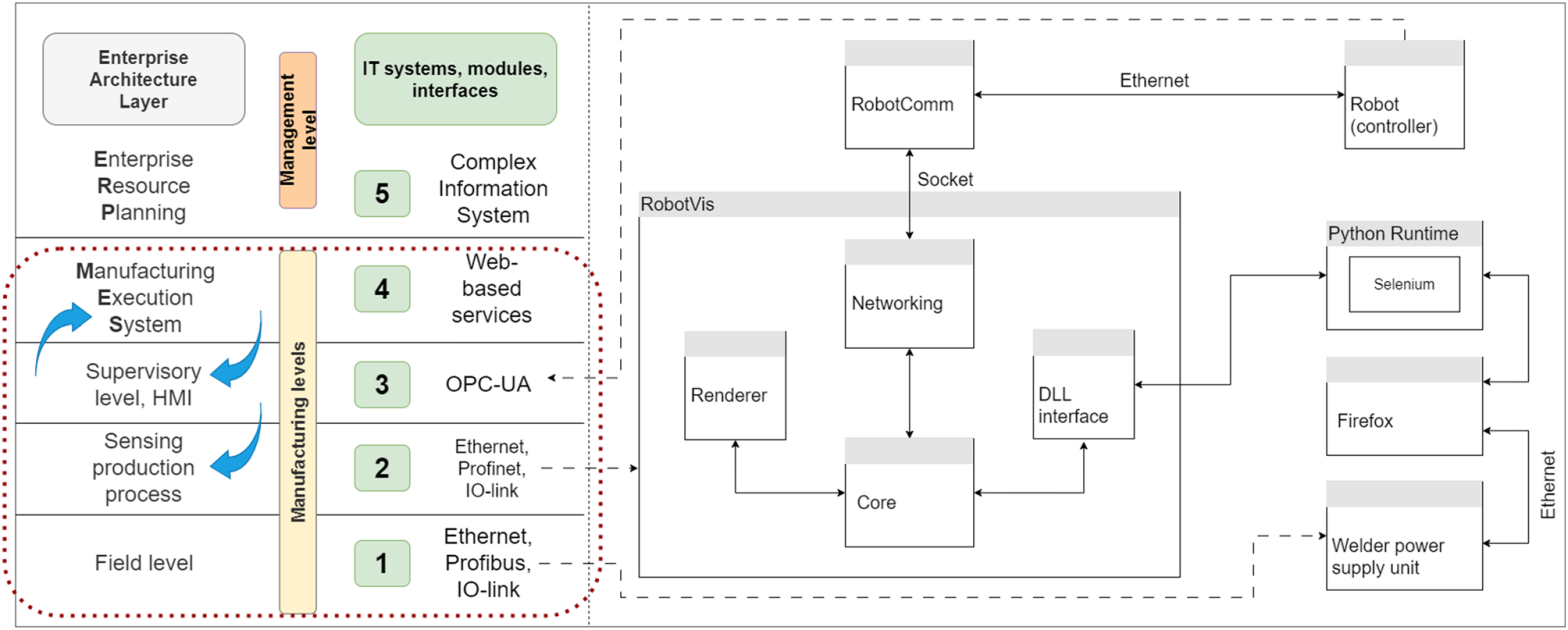
Integration of IT components into the automation structure.

The following architecture was given as an example of how to build the system. The diagram of the Fig. 3 can be used to present the ERP-MES levels already mentioned in the introduction. The area of each level of the production system is indicated by a dotted line since the production processes are displayed at these levels. Elements of the IT architecture that have program functions were identified at the design stage. These are the RobotVis and RobotComm applications. The connection of the IT elements with the relevant levels of the automation architecture is as shown in the figure. Thus, the Robot as a tool attaches the information from the field and sensing levels back to the supervisory level.

The RobotVis program aggregates all the data, can create the record, and also performs the visualization of the current state. Later, the replay of the recorded data can create new opportunities for optimization. The possibilities are also expanding along the lines of cost efficiency. To design the test system, the first step was to design the connectors. The control of the robot can be connected to a PC via a network cable. The following types of devices were used to assemble the system:

- The robot arm *Yaskawa AR1440—Article number: 9519201* organised in cells; two-axis rotator: *Yaskawa MT-1-250S2D Article number: 124614100*
- Welding machine: *Fronius TPS400i—Article number: 4,075,179*

Both the robot and the welding power source use standard ethernet and TCP/IP protocols respectively. Finally, by using a switch and configuring the devices, an external network was set up through which both the power source and the robot were able to communicate with each other. In contrast, an external computer was able to communicate with them. This made it possible to read the motion parameters from the welding robot in real time. Which are the three (actual) motion coordinate values (x, y, z) and the rotation values (Rx, Ry, Rz) along the axes associated with them. To connect the power source of the welder to the PC, it was also necessary to use a power distribution unit, as there were not enough connection points. On the display of the equipment, the values to be applied during welding can be selected as templates to be followed during Windowsing process and digitally recorded in the log file being created. The output signals include, in addition to current and voltage, welding speed, wire feed rate, and the time elapsed since the robot controller was started. Another important piece of information is the active/passive index of the actual welding process, i.e. whether welding is taking place or not.

### Create a virtual robot (preparation of digital twin)

**Figure 4:**
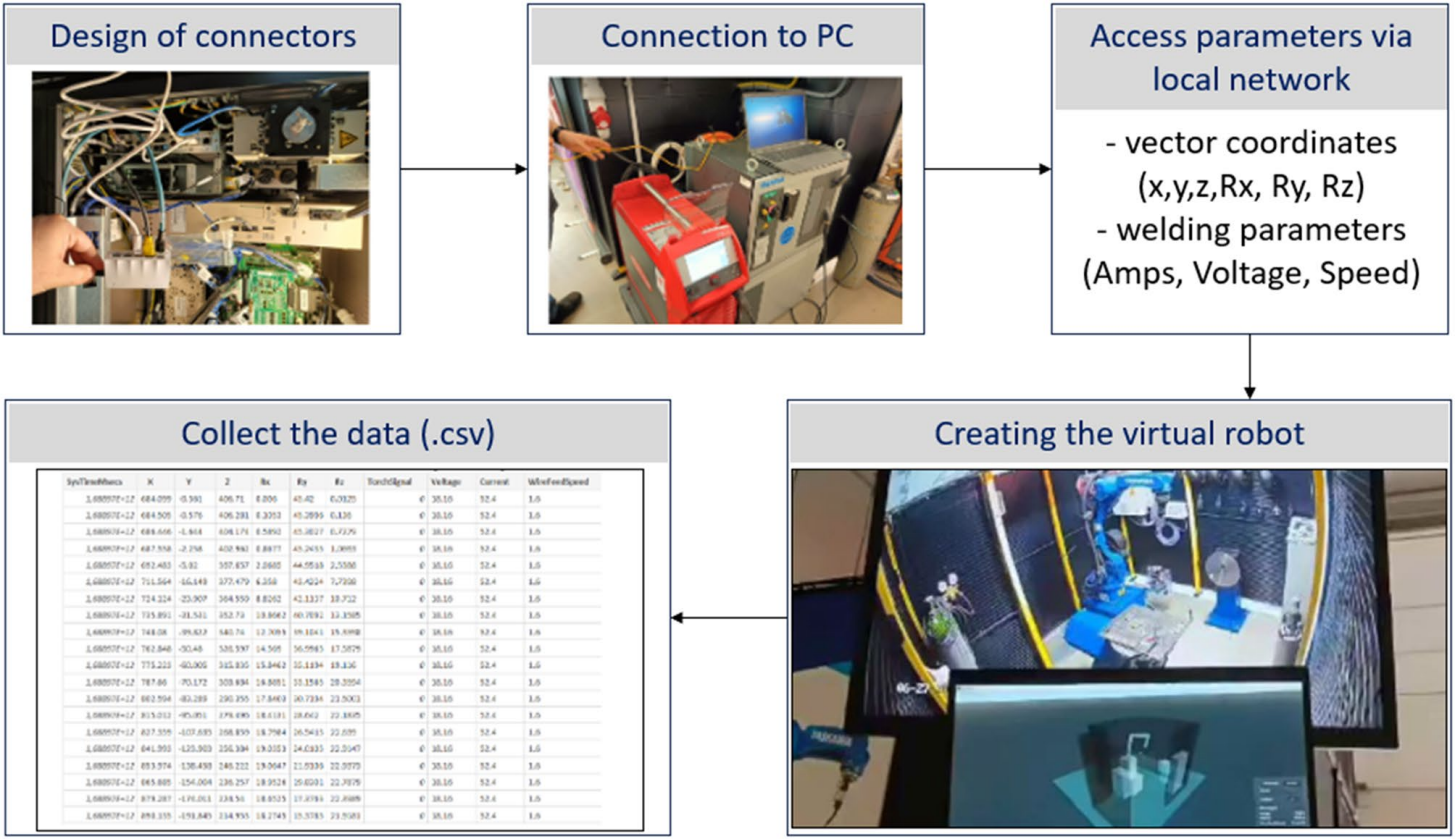
System configuration process.

After aggregating the data, it is possible to create the initial steps of the digital twin. This required a software package that could handle windows and graphical user interfaces, display 3D models, and communicate via socket. A framework for this purpose was already under development at the university and was chosen. Since a dynamic library *.dll* loader module and a python interpreter package linked through it were available, the development time could finally be accelerated. The complete system finally consists of two separate programs, named RobotComm and RobotVis (Fig. 4). Communication between each subpart of the software stack takes time, so it was important to create the simplest architecture possible.

**Figure 5:**
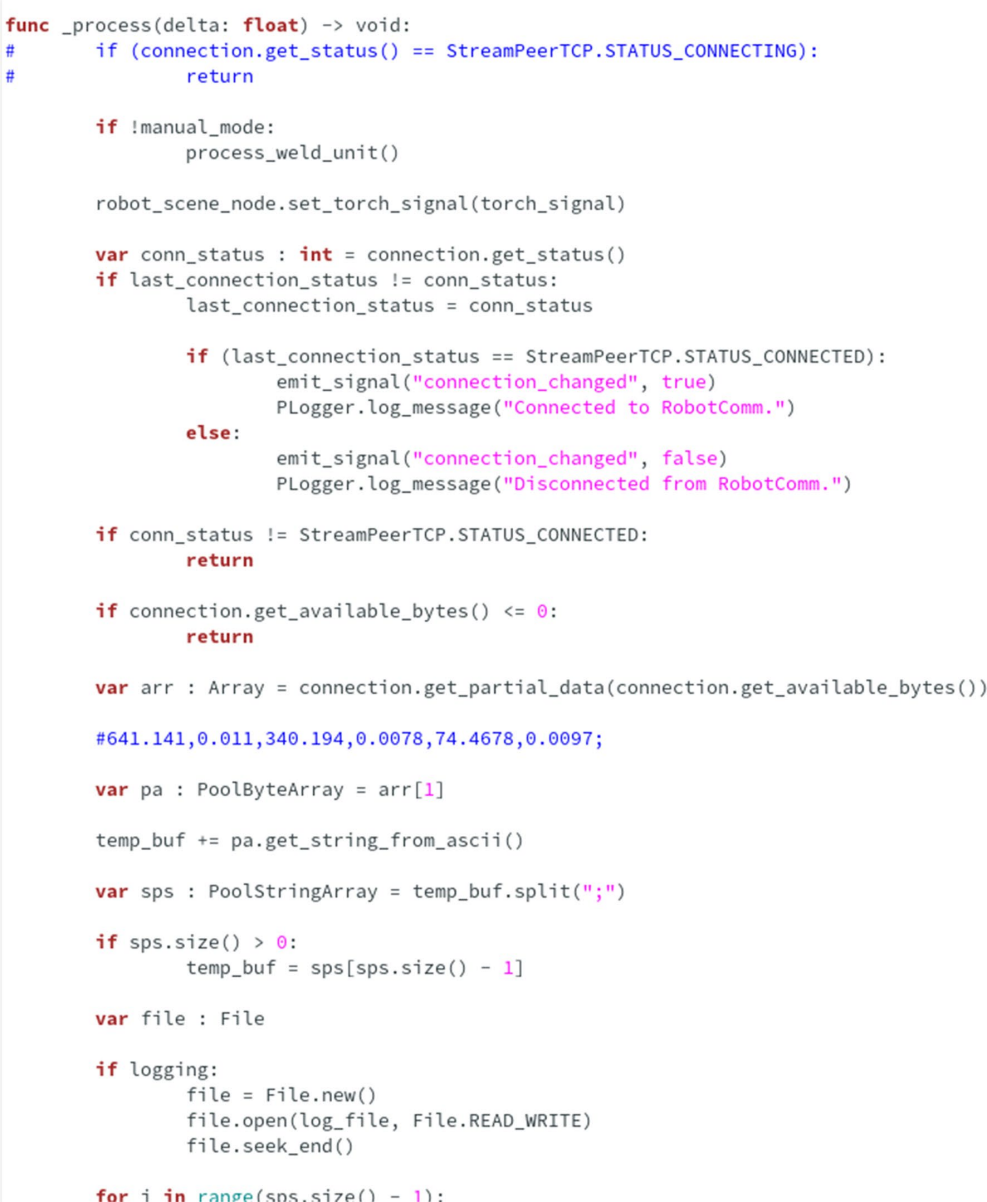
Collect the data.

Using the operating system’s socket programming interface, the RobotComm program links the robot to the RobotVis program (Fig. 5). Which currently only requests and transmits position data. Ideally, this functionality would be part of RobotVis, but the communication software library available for the welding robot was developed using the .NET framework and was not currently directly embeddable due to limitations.

**Figure 6:**
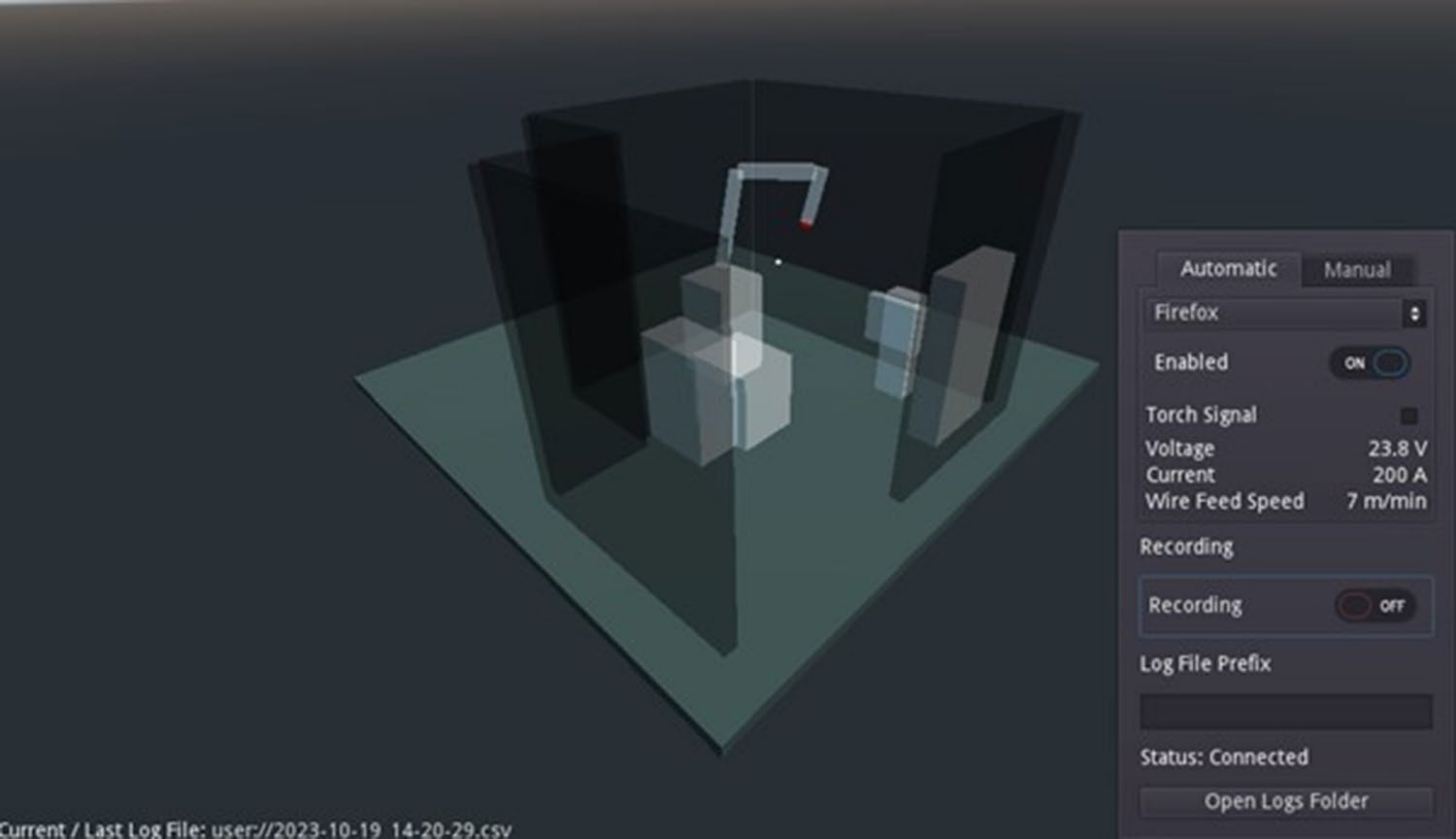
RobotVis software in process.

The main purpose of RobotVis (Fig. 6) is to aggregate, process and display data. After start-up, it automatically establishes a connection with RobotComm and, on user request, with the welding power source. From here, the data is read by the unit’s built-in web interface, which automatically updates the values displayed in the browser via WebSocket. A direct re-implementation of this communication was planned, but a solution with less error potential was (temporarily) developed. Currently, the data is read by a browser test and automation package called Selenium, using a Python interpreter coupled with the dynamic library loader functionality of the operating system.

### Recording of DATA

Data collection and recording was done in files with the extension *.csv*. When the TorchSignal changes from 0 to 1, this is when the actual seam creation takes place. After comparing several *.csv* files, a group repetition of the data series is shown, selecting a recommended welding template, and the recorded parameter series periodically show characteristic properties. The recorded *.csv* files consist of 11 columns of nearly 1000 rows each, with a size of 100 kbyte (Table 1).

**Table 1:** Data table generated from the measurement data.

SysTimeMsecs	X	Y	Z	Rx	Ry	Rz	TorchSignal	Voltage	Current	WireFeedSpeed
1.68897E+12	912.974	− 673.812	− 42.777	0.4746	− 5.7335	− 1.7235	0	18.16	52.4	1.6
1.68897E+12	913.685	− 674.094	− 49.697	0.4565	− 5.6969	− 1.7499	0	18.16	52.4	1.6
1.68897E+12	914.023	− 674.241	− 53.354	0.4476	− 5.6748	− 1.7671	0	18.16	52.4	1.6
1.68897E+12	914.052	− 674.246	− 53.679	0.4462	− 5.6725	− 1.7677	0	18.16	52.4	1.6
1.68897E+12	914.052	− 674.246	− 53.679	0.4462	− 5.6725	− 1.7677	1	18.16	52.4	1.6
1.68897E+12	914.017	− 674.245	− 53.677	0.4454	− 5.6725	− 1.7669	1	18.16	52.4	1.6
1.68897E+12	913.958	− 674.249	− 53.679	0.4457	− 5.6724	− 1.7669	1	18.16	52.4	1.6
1.68897E+12	913.849	− 674.253	− 53.683	0.4462	− 5.6725	− 1.7676	1	18.16	52.4	1.6
1.68897E+12	913.645	− 674.259	− 53.686	0.4463	− 5.6724	− 1.7674	1	18.16	52.4	1.6
1.68897E+12	913.528	− 674.257	− 53.689	0.4462	− 5.6719	− 1.7672	1	18.16	52.4	1.6
1.68897E+12	913.429	− 674.272	− 53.674	0.4458	− 5.673	− 1.7678	1	18.16	52.4	1.6
1.68897E+12	913.33	− 674.269	− 53.683	0.4458	− 5.6724	− 1.767	1	18.16	52.4	1.6
1.68897E+12	913.234	− 674.275	− 53.685	0.4464	− 5.672	− 1.7684	1	25.29	67.1	1.38
1.68897E+12	913.201	− 674.267	− 53.681	0.4463	− 5.6724	− 1.7669	1	25.29	67.1	1.38

In total, 150 parts and 450 seams were produced, which means 150 files (*.csv*) with all the associated data:

- The time elapsed since the system was switched on;
- Coordinates of movement—6 columns;
- Process of welding (yes/no);
- Welding parameters—3 columns.

## Discussion

**Figure 7:**
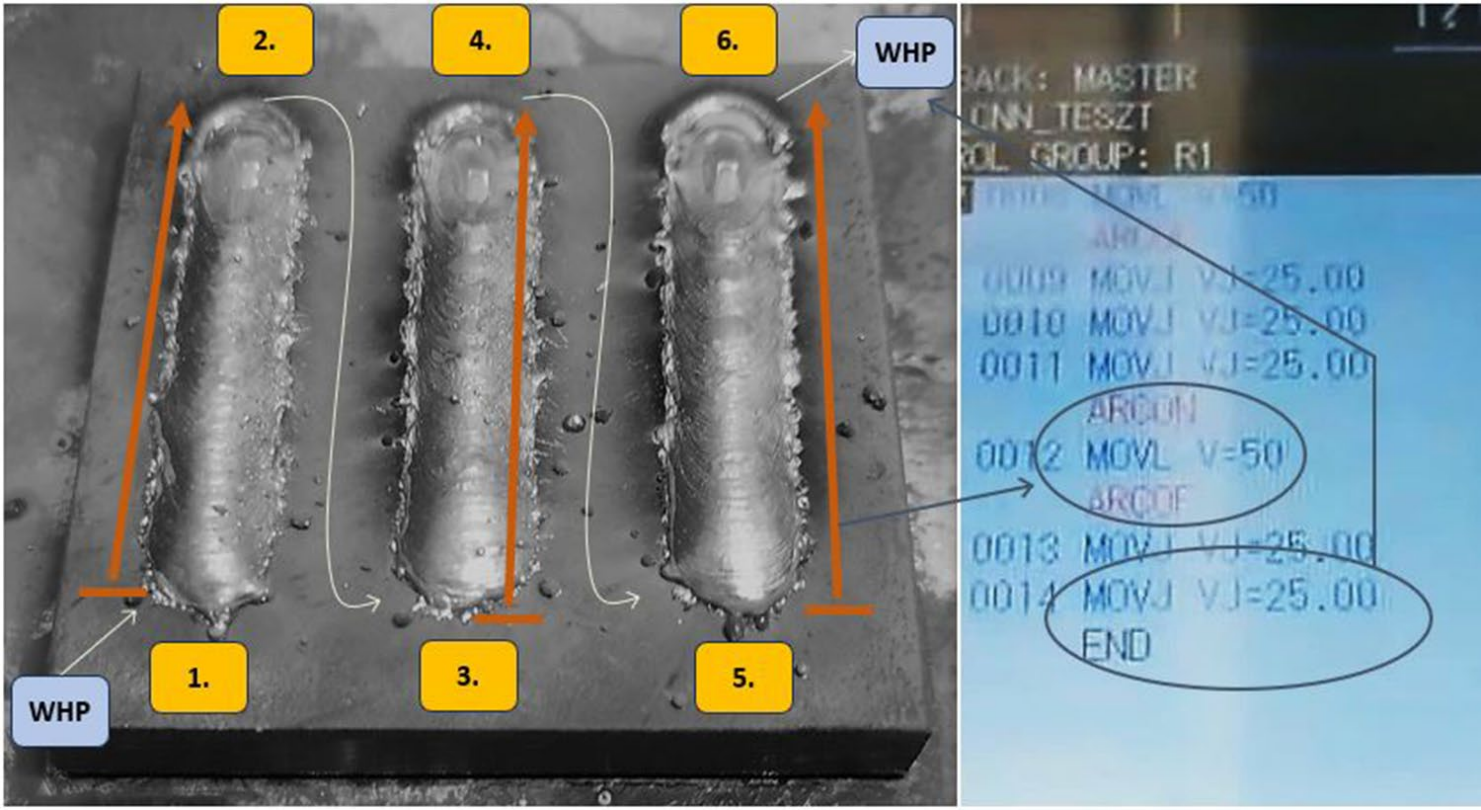
Test piece with seems.

A small number of welding trials were carried out, producing 150 parts, with 3–3 identical welds per part. Figure 7 below shows one of the 150 test pieces with the weld direction marked. The workpiece is a 50 mm × 50 mm flat iron with a plate thickness of 6 mm. The welds are cobweb welds with different parameters fixed in a table. The welds are 40 mm long. Three welds with the same parameters are shown on one weldment. During the analysis, have been compared the related data recorded during the seam formation of the workpieces located at the same location value (first–first components of group 30). For the seams of these completed workpieces, only the welding speed changed, but not the initial values of the voltage and current (18 V and 40 A). The experiment is a preliminary experiment of a (later) system monitoring and system optimization framework that looks for outliers and other unexpected events. This prepares the intervention tasks of the process control framework using digital twin (supervision + independent intervention).

As shown in the figure, the orange arrows indicate the direction of the seam preparation and then the jump to the first position of the next seam. Next to it, only a part of the robot program is shown (finishing routine) The weld is interpreted as a path segment between ARCON-ARCOF called macros. At the beginning and end of the program, a jump to the WHP (Work Home Position) position is defined.

By increasing the welding speed stepwise (between 30–40–50–60–70 cm/min), 5 groups were formed, the other parameters being synergistically adapted to the others:

- The welding voltage is between 18 and 23.8 V;
- The current magnitude between 40 and 200 A
- The wire feed rate varied between 1.2 and 7 m/min.

**Figure 8:**
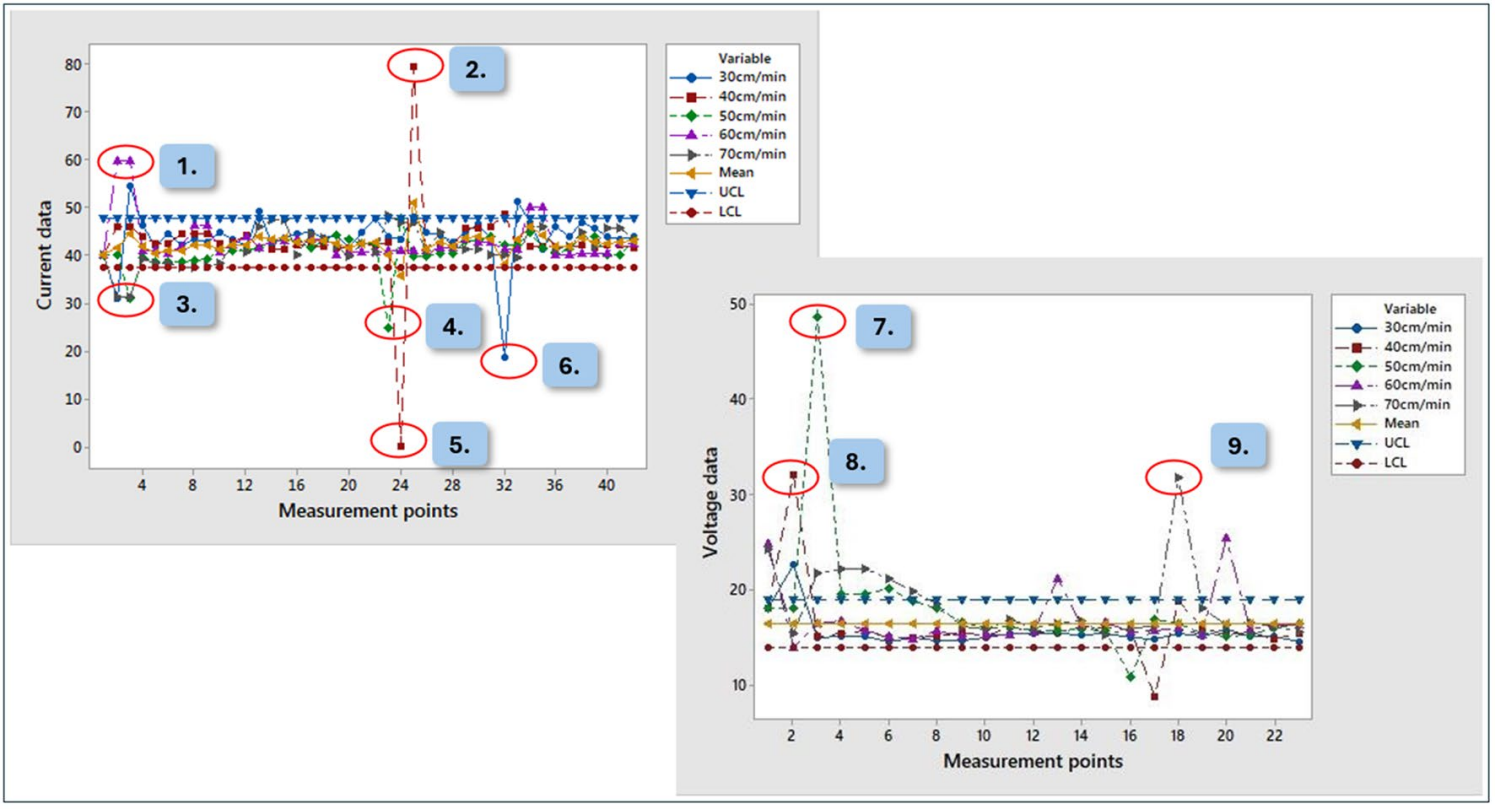
Evaluation of data of current and voltage.

Looking at the voltage values, the diagram (Fig. 8.) clearly shows that most of the fixed values are between the recommended lower and upper control limits (red line/LCL and blue line/UCL). The recommended average value line (green), due to extreme outliers, is shifted to a value higher than half of the measured values. The voltage value of 18 Volt, set on the power source, is between the calculated upper limit and the recommended average value (yellow line) during the welding process. Looking at the current on the diagram clearly shows that the recommended lower and upper control limit (red line/LCL and blue line/UCL) and the recommended mean value line (yellow line) are shifted downwards due to outliers, compared to the majority of the data recorded. At the 40 Ampere current set on the power source, higher values are seen in most cases during the welding process. The charts were created with *Minitab 17* statistical software. The individual curves thus show values recorded under the current welding speeds, made with the smallest input welding parameters per group (18 Volts and 40 Amps), recorded during welding processes. In the case of a given seam, from the beginning of welding to the end of welding, the recorded values are superimposed. Examining how the value of the applied input parameters changes when the welding speed is changed. Overall, as a result of the comparison, it can be seen that there are outliers at the beginning of the seam, as well as in the last quarter of the total length of the seam.

Initially, during the data captures, there was a very rapid increase in the size of the data sets, which after a while became difficult to manage due to their sheer size. This has been caused by the excessive frequency of data recording, so fine-tuning was necessary. It is important that the data logging takes place from the actual start of the welding robot, and that no logging takes place until the robot moves. Also, it had to be solved so that the data recording could be started and stopped within the program. If there is a wait while the program is running, logging is not necessary (if the movement coordinate values do not change, the robot arm is stationary). The logged lines have been recorded in a *.csv* file, but to accurately reconstruct the access path from the data, it was also necessary to record the elapsed time. After the connection is established between the PC and the robot, the data recording has been stable, with no noticeable downtime.

## Summary

In our study, a virtual platform was created. As a further development of this theme, a digital twin can be implemented, which already forms a complex monitoring system. A framework has been prepared, the input parameters of which are as follows:

- On the one hand, the coordinates of the movement of the six-axis welding robot;
- On the other hand, the parameters generated by the welding equipment connected to the robot during seam formation (voltage, current, welding speed, wire feed speed).

**Figure 9:**
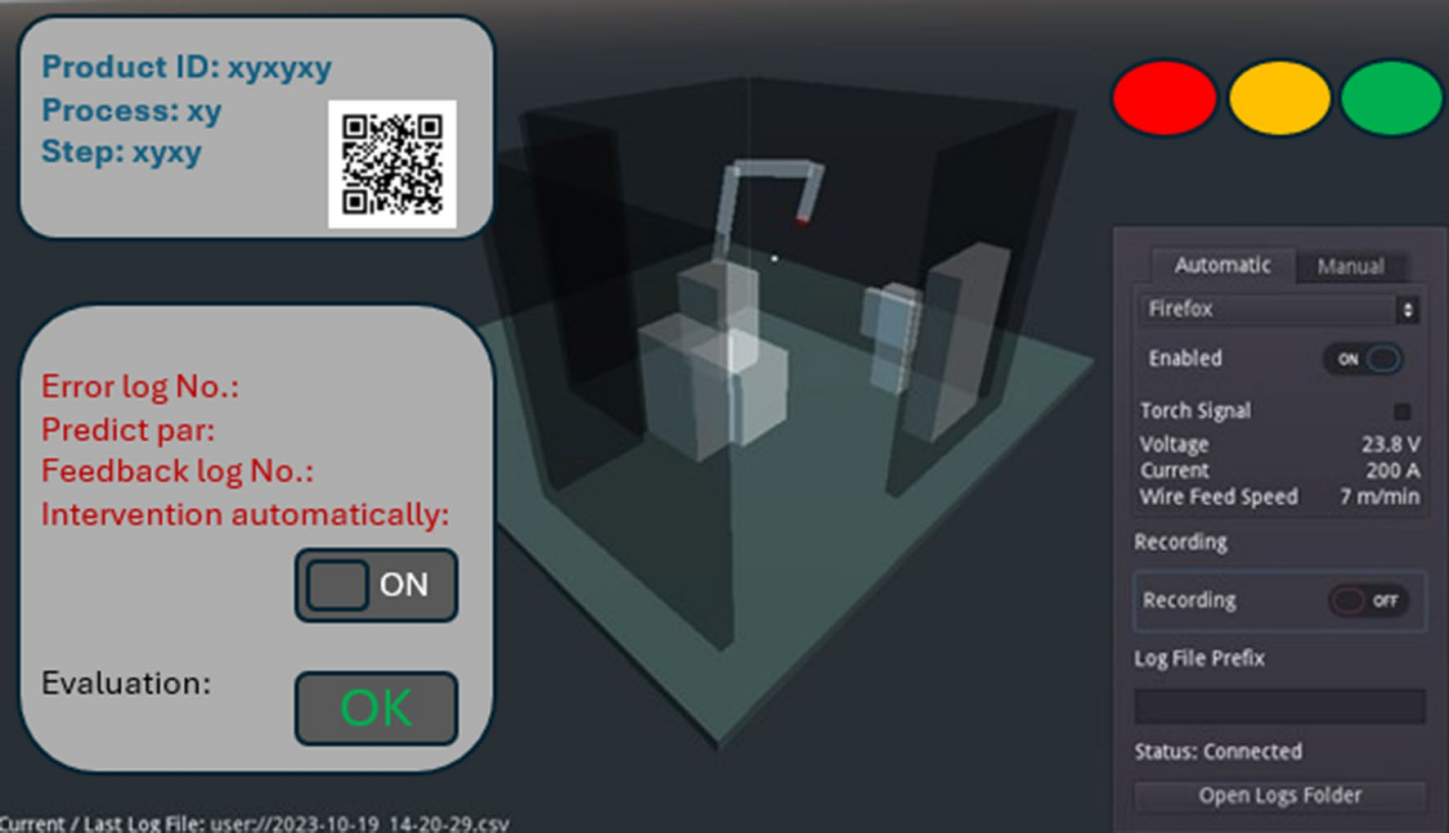
A possible digital interface design example for the use of digital twins.

Taking into account the changes in these parameters, the virtual robot moves in parallel, and the recording of the digital content also starts. Documentation of the seams created during the welding experiment thus enabling the comparison of workpieces in the same position according to the experimental table. According to the experience, the device has been available by one connection point (connecting locally shared—WIFI). One machine is served by the framework, no group connection is possible. Can able the capturing data and display the current movement. The recorded data sets also provide the possibility to independently check with coordination parameters. After running the same program sequences, the geometrical parameters do not return the same values and should be calculated with minimal differences. The example in Fig. 9 shows an idea of how the digital twin can be applied. For example, based on the outliers in diagrams, if they actually represent a technical failure and not, say, a faulty data recording, then the process data can be changed by feeding them back. It is important to note that no matter how powerful the neural network behind such a system is, it should not be left to algorithms alone to make the decision, and human activity in this should be maintained. However, as shown in the figure, it is worthwhile to keep a switch on by invoking automatism. A clear visual andon status indicator is displayed on the monitor to assist the operator, red: NOK, orange: not ready, green: OK.

The following optimization tasks can be performed by assembling the system, which is also the way forward for this research work:

- *Real-time system monitoring in CAW:* The immediate evaluation of product data can only be achieved if there is a maximum of 1–2 s between the time of the production process steps and the time of the monitoring displays. The task is to monitor the process result and to monitor and control changes in the input data.
- *Estimation of optimal parameters for a welding task:* In welding, as in any other technology, the correct choice of technological parameters is key. In implementing digital manufacturing, the optimal choice of parameter window becomes possible if the algorithms implementing the decision-making process can reliably manage the technological processes. The challenge is to create an algorithm to support the decision-making and to improve the evaluation.
- *Seam sequence optimization:* When welding technologies are used, the material structure around the welds formed as a result of the thermal processes is transformed. The changes in material structure often cause an increase in the stresses in the material structure by increasing the lattice defects and thus the warpage of the welded pieces. To control these warpages, technologists use seam sequence determination, which can be modelled in advance in such digital twin applications.
- *Simulation of welding trajectory:* In-process inspection is already included in the toolbox of most off-line programs. However, the focus of future IT development on this topic will certainly be on the ability of the framework to provide immediate re-plannability in the light of individual production events, i.e. whether the algorithms have the reliability of evaluation to deliver the optimal production plan with certainty.

During the data collection, the established framework recorded outliers in addition to the expected values. This shows that in reality, these processes need further investigation/system monitoring. With the further development of the digital twin, we intend to direct the research towards the management/intervention of outliers and process optimization.

In addition to the identified development directions, there may be other areas of research that are not yet visible. Our experience is that with each research project, new and new lines of thought can be started, as digitalization evolves with us. Our task is to integrate new results into technological processes to achieve the required efficiency.

## Data Availability

The datasets used and/or analyzed during the current study are available from the corresponding author upon reasonable request.
